# Definition, Fabrication, and Compression Testing of Sandwich Structures with Novel TPMS-Based Cores

**DOI:** 10.3390/ma17215150

**Published:** 2024-10-22

**Authors:** Alexandru Vasile, Dan Mihai Constantinescu, Iulian Constantin Coropețchi, Ștefan Sorohan, Dragoș Alexandru Apostol

**Affiliations:** 1 Department of Strength of Materials, National University for Science and Technology POLITEHNICA Bucharest, Splaiul Independeţei 313, 060042 Bucharest, Romania; alexandru.vasile@mta.ro (A.V.); iulian.coropetchi@mta.ro (I.C.C.); stefan.sorohan@upb.ro (Ș.S.); dragos.apostol@upb.ro (D.A.A.); 2 Faculty of Aircraft and Military Vehicles, Military Technical Academy “Ferdinand I”, G. Coşbuc Blvd. 39–49, 050141 Bucharest, Romania; 3 Technical Sciences Academy of Romania, Dacia Blvd. 26, 030167 Bucharest, Romania

**Keywords:** TPMS, implicit modeling, sandwich structures, metamaterials

## Abstract

Triply periodic minimal surfaces (TPMSs) constitute a type of metamaterial, deriving their unique characteristics from their microstructure topology. They exhibit wide parameterization possibilities, but their behavior is hard to predict. This study focuses on using an implicit modeling method that can effectively generate novel thin-walled metamaterials, proposing eight shell-based TPMS topologies and one stochastic structure, along with the gyroid acting as a reference. After insights into the printability and design parameters of the proposed samples are presented, a cell homogeneity analysis is conducted, indicating the level of anisotropy of each cellular structure. For each of the designed metamaterials, multiple samples were printed using a stereolithography (SLA) method, using a constant 0.3 relative density and 50 µm resolution. To provide an understanding of their behavior, compression tests of sandwich-type specimens were performed and specific deformation modes were identified. Furthermore, the study estimates the general mechanical behavior of the novel TPMS cores at different relative densities using an open cell mathematical model. Alterations of the uniform topologies are then suggested and the way these modifications affect the compressive response are presented. Thus, this paper demonstrates that an implicit modeling method could easily generate novel thin-walled TPMSs and stochastic structures, which led to identifying an artificially designed structure with superior properties to already mature topologies, such as the gyroid.

## 1. Introduction

Sandwich structures with metamaterial cores have become increasingly popular among researchers, and various types of topologies have been proposed. The traditional method of developing mechanical metamaterials involves a heuristic approach, where architected materials are manually developed and tested, in order to be confirmed or disproved as being a viable option for certain implementation directions. This also gives rise to more or less intuitive rules regarding further optimization.

The lack of restrictions when designing sandwich structures gives rise to an endless combination of existing or imaginable core geometries, sheets, interfaces, materials, and fabrication technologies. This design freedom with impressive proportions can be seen as an advantage due to the prospect of defining a metamaterial with superior properties to those already researched, but it can also prove to be a disadvantage, as it becomes overwhelming to focus on a clear direction and explore it. The following prospects have been taken into account to help guide the choice of a type of metamaterial to be used as a sandwich core.

The endeavor is to consider a novel topology or an existing geometry that has generated interest in current research. Such a geometry should be clearly defined by computer-aided design (CAD) models and/or mathematical descriptions, it should be possible to manufacture through conventional or additive manufacturing processes that do not involve changes to the nominal geometry, and it should be easy to scale and parameterize. Also, it should be tested experimentally, finite element simulations should be feasible and, finally, the production costs should be affordable.

The current literature offers many valuable observations regarding the general mechanical properties, failure modes, and how different parameters influence the mechanical performance. From structures with constant cross-section metamaterials, such as honeycombs or chiral, to three-dimensional topologies, such as truss-based, foam, corrugated, or honeycomb cores, all had a long exposure to research and implementation in practical applications. However, with the progress of additive manufacturing technologies, very complex topologies can now be generated and analyzed with methods that involve an automatic approach. A more detailed classification of the existing types of sandwich structures with metamaterial cores is presented in [1,2,3].

The type of metamaterial that blends all the criteria stated above is the TPMS structures. They display a periodic geometry that presents wide parametrization possibilities and have advantages in the complete elimination or minimization of support structures needed during additive manufacturing processes [4]. They are easy to define and configure, especially using an implicit modeling approach, while being able to occupy any given domain due to the continuous nature of their consisting surfaces. At the same time, their minimal surface ensures material efficiency by reducing unnecessary mass, and there are indications that such topologies have better loading capacity compared to traditional truss-based lattice structures, where ligament intersections act as stress concentrators [5,6,7]. Furthermore, TPMS geometries have not been intensively studied, except for well-established geometries such as the gyroid [8,9], Schwarz [10,11], Diamond [12,13,14], Lidinoid [15], Split-P, [16], and Neovius [17,18].

Choosing the relative density value is also important as it has been shown that there is a transition zone between layer-by-layer and wall-fracture failure. Observations in [19,20,21] can provide important insights into obtaining a more uniform deformation mode without premature fracture even at high strain values.

Systematic studies of different mechanical properties of the most common TPMS structures, such as [22,23,24,25], can provide valuable indications on possible engineering applications of such topologies.

Tailoring the mechanical response of TPMS structures, such as the energy absorption capacity and general stability of the structure, can be done by modifying the dimensions and orientation of the periodic cell or employing a functionally graded design. Relevant articles useful for understanding how different geometric alterations of the uniform design affect the mechanical properties are [12,13,26,27].

Additive manufacturing (AM) techniques, such as selective laser melting (SLM) [28,29], direct metal laser sintering (DMLS) [30,31], and stereolithography (SLA) [32,33], have already been demonstrated to allow for precise creation of complex TPMS topologies, from metals, polymers, and ceramics. Overviews of the fabrication parameters of each method have been put together in [34,35]. Ongoing research aims to refine fabrication parameters to achieve higher resolution, reduce defects, and improve mechanical properties, while also investigating new materials and hybrid AM approaches to enhance the performance and scalability of TPMS structures.

The present study establishes the mathematical formulations of eight novel proposed TPMS topologies to be compared to the gyroid structure. In addition to this, a stochastic topology is also generated. The procedure for printing and post-processing the samples is further described. Next, a geometry analysis is performed for the proposed topologies and their level of anisotropy is analyzed. Compressive testing of the proposed structures is conducted, and different types of deformation modes are identified, separated as “bending-dominated”, “stretching-dominated”, and “mixed” behavior. Using experimental data, the effective Young’s modulus and yield stress for different relative density values were estimated. A discussion and conclusions emphasize that an implicit modeling method can easily generate novel thin-walled TPMS and stochastic structures for specific engineering applications.

The next steps are to develop a method that can easily integrate implicit modeling designs in finite element analyses and compare the simulation results with experimental data. Also, the proposed samples will be subjected to low-velocity impact testing. Another direction is to automate the design generation of such topologies, given a list of the constraints imposed by the fabrication method and their observed mechanical behavior.

## 2. Materials and Methods

### 2.1. Topology Definition

**Figure 1:**
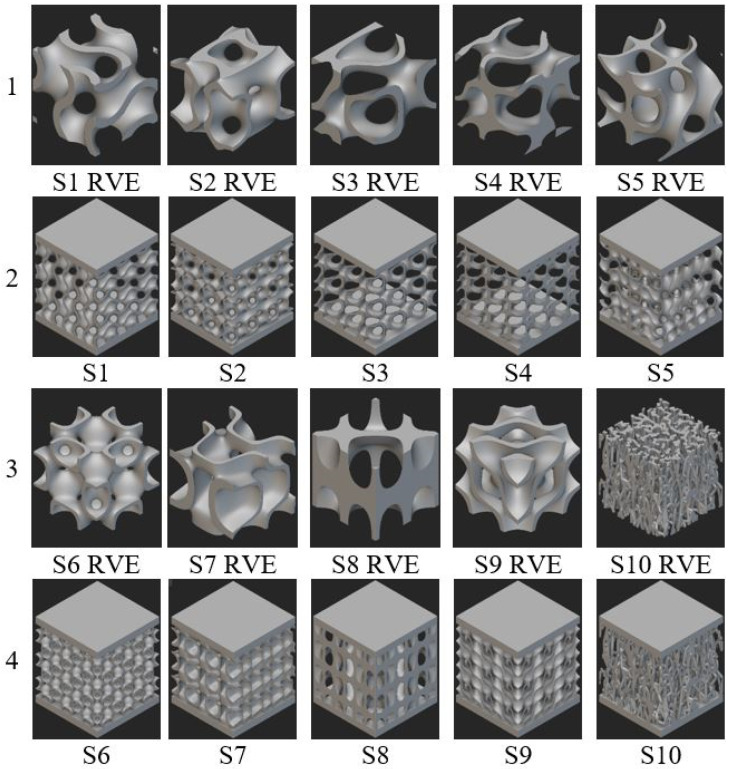
Rows 1 and 3: proposed RVEs. Rows 2 and 4: sandwich structure containing 27 RVEs. Notations for sample topologies correspond to those from Table 1.

The mathematical functions that describe the geometries of the artificially architected metamaterial structures considered in this work are presented in Table 1. Equation (1), established initially by Schoen [36], describes the most common type of TPMS structure, the gyroid cell, used in this research as a benchmark representative volume element (RVE), being an already well-established geometry. Equations (2)–(9) show heuristically determined mathematical functions, suitable for obtaining continuous, non-intersecting, self-supporting architectures. A stochastic geometry, notated as S10 in Figure 1, was also obtained by using NTopology software v4.5.3, simulating a foam-type infill, by imposing the following conditions: the struts follow the perpendicular direction between the two sandwich sheets, with an average distance between the ligaments of 3.5 mm and an average number of 6 struts intersecting at the same point.

The analyzed samples were designed as sandwich structures incorporating metamaterial cores, shaped as a cube with 30 mm sides, positioned between two 3 mm sheets. To maintain a constant relative density of 0.3 across all samples, the thickness of the walls was adjusted accordingly, and, since the minimum printing resolution of the chosen manufacturing method is 50 µm, only multiples of this value were considered. The value for relative density was selected with regard to the indications in the relevant literature, in order to achieve a homogenous, layered deformation, without premature cracking of the walls [19,20].

To define the metamaterial volume, two surfaces are arranged symmetrically with respect to the value for which *f*(*x,y,z*) = 0, and the volume contained between them defines the RVE, shown in Figure 1, rows 1 and 3. Rows 2 and 4 display the cellular structure incorporating 3 × 3 × 3 RVEs for each sample. An implicit modeling method was employed to create the samples. Both NTopology and TPMS Designer library v3.2.1.2 from Matlab were used, in order to verify the accuracy of the results. When generating the STL file format used in preparing the manufacturing process, the same resolution of 50 µm was applied.

### 2.2. Fabrication Method

The interdependence between a part’s geometry and its printability is, apparently, easy to identify, but there are certain printability indications that can prove critical when designing cellular structures with complex geometries. A complicated formulation of the topology can generate inaccessible areas, where the presence of supporting elements would lead to the modification of the geometry and to results that do not faithfully characterize the proposed model. Also, resin and powder-based fabrication methods are sensitive to closed enclosures, because material remains captive inside, not being able to be removed during post-processing and resulting in significant changes to the geometry. The presence of geometric elements characterized by right angles constitutes areas where the printed sample cannot support itself and lead to an increase in the number of additional supports required for manufacturing, and to an increase in the risk of the layers exfoliating. This negatively influences the print and post-processing times, increases the risk of surface defects and material consumption and can lead to significant flatness deviations. In addition to visible defects on a macroscale (cracks, deformations), microscale defects may also appear, thus requiring specialized equipment to be used.

To overcome these limitations, an additive fabrication method with very high resolution was needed, that would require minimal post-processing. Plus, minimization of surface defects and internal porosity was desired in order to faithfully capture the mechanical response of the proposed structures. Thus, samples were fabricated through an SLA method, using a Form 3L printer (Formlabs, Somerville, MA, USA), a 50 µm layer thickness, 50% density for lateral supports, and a 0.35 mm contact area between the sample and support, to facilitate subsequent removal without damaging the surfaces. The material used to fabricate the samples is a photopolymer resin, Tough 1500 v1 (Formlabs, USA), developed to provide a balance between stiffness and strength. The post-cured average mechanical properties established by us through tensile testing are as follows: 34.47 MPa ultimate tensile strength, 1.29 GPa tensile modulus, and 26% elongation at break. Their determination is not a subject of this paper.

**Figure 2:**
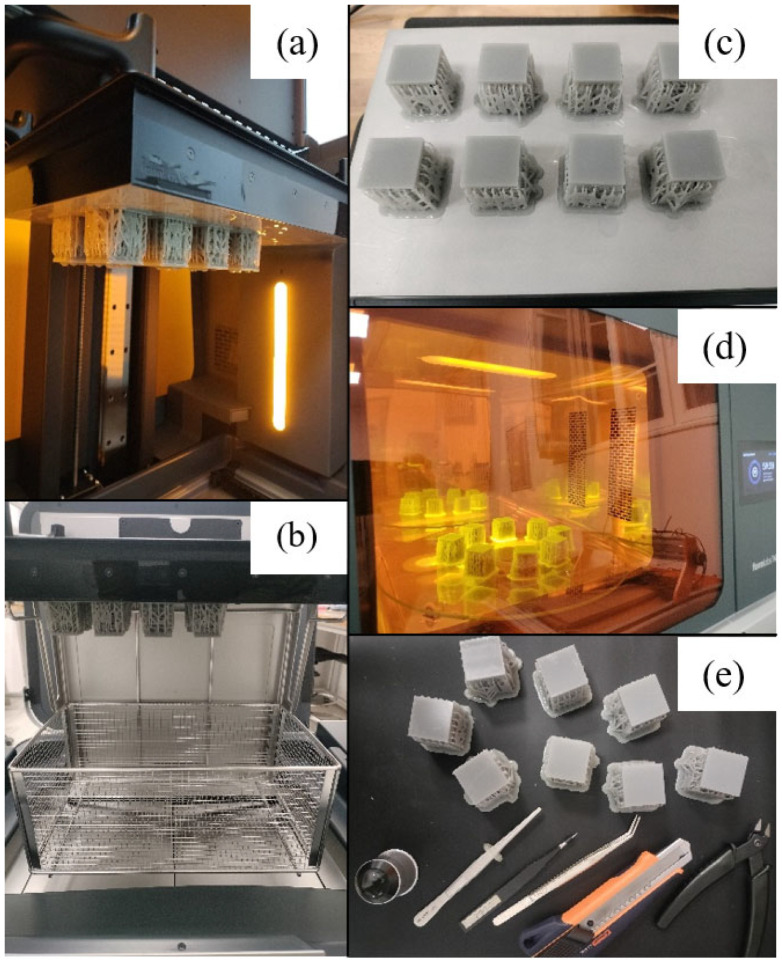
SLA manufacturing process: (**a**) sample printing; (**b**) post-processing by washing with isopropyl alcohol; (**c**) samples removal from the printing table; (**d**) post-processing by ultraviolet radiation treatment; (**e**) manual removal of lateral supports.

The procedure for printing and post-processing the samples is briefly described below and is highlighted in Figure 2. The predefined layer-by-layer printing program is used by the printing station, where the samples are printed (Figure 2a). In order to avoid errors generated by human intervention, post-processing was automated as much as possible. It consists of two steps. Initially, samples are inserted into the Form Wash L unit, shown in Figure 2b, which automatically stirs the isopropyl alcohol to remove the excess resin that has adhered to the parts’ surface. After the samples are removed from the printing table, as shown in Figure 2c, they are placed inside the Form Cure L treatment unit (Figure 2d), where they are cured at a temperature of 70 °C and subjected to ultraviolet radiation with a wavelength of 375 nm for 1 h. At the end, the lateral supports are manually removed (Figure 2e), totaling an estimated production time for one sample of 5 h, and a production cost of 4.7 EUR/sample. While the cost has a linear evolution as related to the number of printed parts, the fabrication time drops to an estimated 2 h/part when 10 samples are printed.

### 2.3. Geometry Analysis

The first step was to verify that the mean surface curvature of each of the samples proposed is zero, thus respecting the curvature requirements specific to TPMS-type geometries.

**Figure 3:**
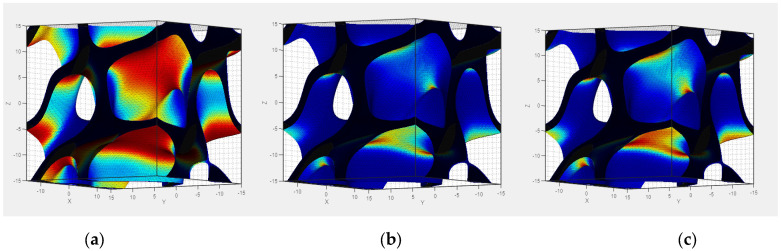
Representation of supports needed during additive manufacturing for different technologies: (**a**) FDM, (**b**) SLA, (**c**) PBF.

In order to obtain estimative information regarding the occurrence of errors during the manufacturing process, the TPMS Designer library for Matlab was used, as discussed by Jones et al. in [37]. This provides indications on the critical areas in which defects can occur during printing, taking into account the average curvature of the surfaces correlated with the need for supports for different types of additive manufacturing technologies: material extrusion (FDM), photopolymerization (SLA), and powder bed printing (PBF). As an example, Figure 3 visually presents the result of such an analysis for S4—the red color indicates the possible critical surfaces developed during printing. It can be noted that, although photopolymerization offers the most favorable results, none of the three processes allows manufacturing without the use of external supports. However, considering the multitude of existing printing techniques, each with its own characteristics, it is necessary to verify the validity of these estimates, through experimental testing.

**Figure 4:**
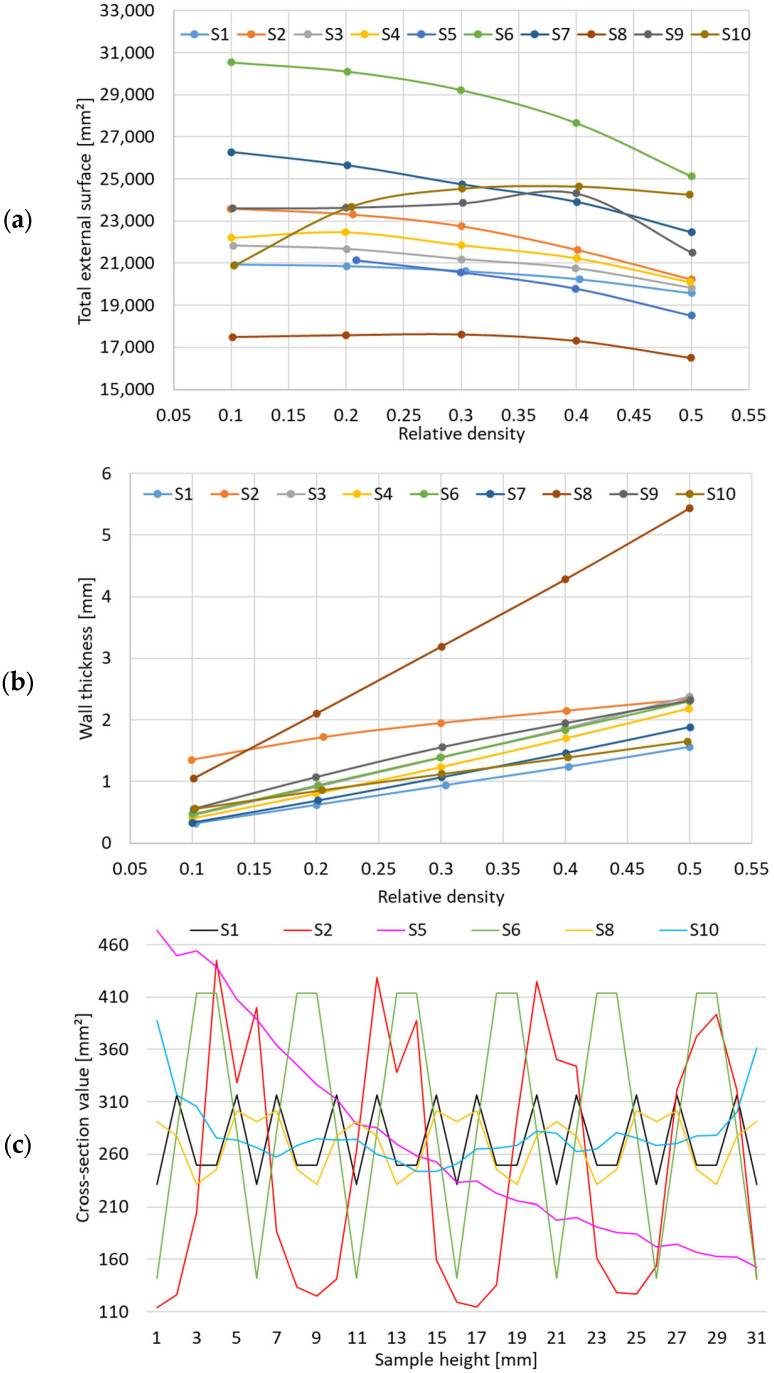
(**a**) Variation in the external surface as a function of relative density; (**b**) variation in the wall thickness as a function of relative density; (**c**) cross-section area value as a function of the sample’s height.

The total external surface of the samples as a function of relative density from 0.1 to 0.5 is shown in Figure 4a. By associating with the model of each representative cell, it is obvious that the smaller the dimensions of the cell features, the higher the value of the total surface.

From this representation, it looks like S6 and S7 are the best candidates for a higher total surface. Considering that many of the imagined structures delimit two separate chambers, a higher value represents an advantage, if applications such as heat exchangers are considered. Similarly, Figure 4b displays the variation in the wall thickness of the samples. An agglomeration of the majority of the samples around the values of 0.5–2 mm is visible over the entire range of relative densities, with only one outlier, S8, which was defined on the basis of thicker walls that delimit internal chambers of bigger volumes.

For an easier visualization of the distribution of the mass inside the samples, the variation in the cross-sectional area was established by slicing each topology with planes parallel to the sandwich sheets, at an interval of 1 mm, along the thickness of the structure. Then, the entire area filled with material was determined, for each generated plane, resulting in the evolutions presented in Figure 4c. It is obvious that the mathematical functions that define the metamaterials offer a wide range of types of geometries, from those with a reduced mass distribution along the *Z* (vertical) axis, such as the gyroid sample S1 or sample S8, to those with large gradients, such as samples S2 and S6; from sample S5, whose area curve shows a controlled decrease due to the wall thickness gradient, to sample S10, stochastically defined, which comes closest to a constant evolution of mass distribution along the thickness of the structure.

Due to the periodic arrangement of the sheet network lattice structures and the implicit model approach used, the resulting samples show anisotropy, with mechanical properties having different values in different loading directions. Thus, it is important to characterize the degree of anisotropy of the newly created geometries, in order to optimize their orientation for different applications. For geometries lacking cubic architecture, a simplified representation of the anisotropy can also be given using the ratio *E*/*E*_max_, where *E* is the local elastic modulus and *E*_max_ is the value of the maximum elastic modulus. With this method, it is possible to visualize the degree of anisotropy of a structure through a 3D graphical representation of the value of the effective Young’s modulus in the three orthogonal main loading directions. In the case of an isotropic material, for which the Zener ratio is 1, this surface is perfectly spherical, suggesting identical mechanical properties in all directions [38]. The degree of anisotropy for the developed geometries was studied using NTopology software.

**Figure 5:**
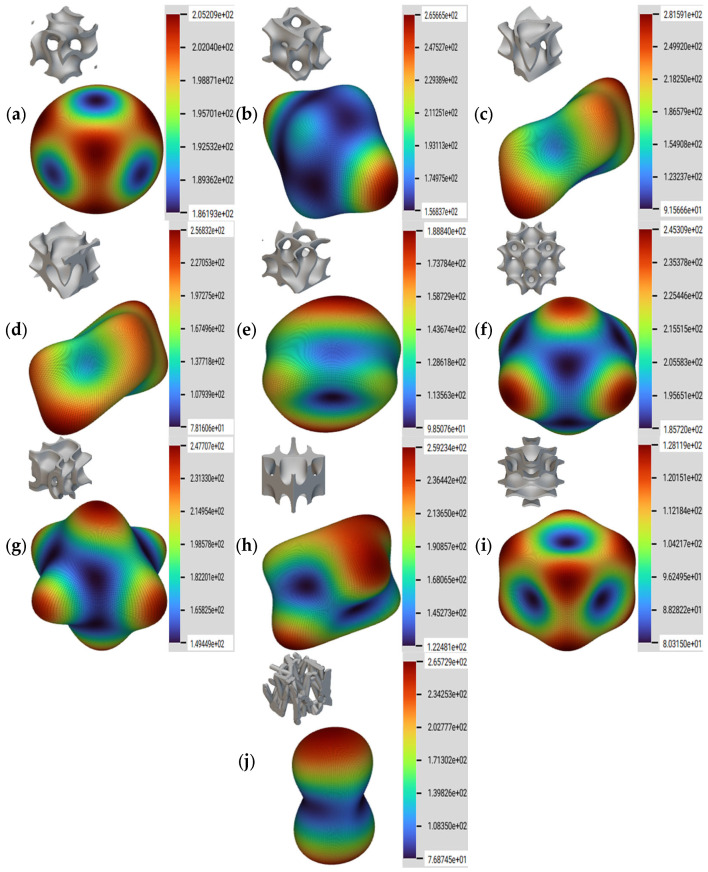
Representation of anisotropy for the proposed samples: (**a**) S1; (**b**) S2; (**c**) S3; (**d**) S4; (**e**) S5; (**f**) S6; (**g**) S7; (**h**) S8; (**i**) S9; (**j**) S10.

The directional homogenization, along with the associated RVE and the legend specifying the value for the Young’s modulus in MPa, are presented in Figure 5. The gyroid, S1, has the shape closest to a sphere; therefore, it has the highest isotropic behavior. Other samples that show a behavior dominated by reduced anisotropy are geometries S6, S7, and S9, with similar modulus values on the main loading directions. The stochastic geometry S10 was analyzed in the entire definition volume and showed a strong anisotropic behavior, with the vertical loading direction being stiffer due to the orientation of the struts.

## 3. Results

### 3.1. Compressive Testing

**Figure 6:**
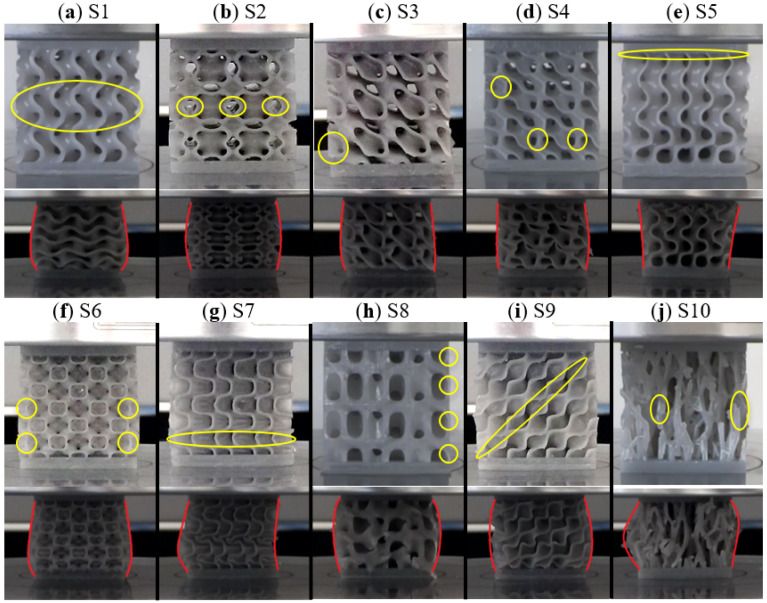
Images taken during compression testing. Yellow curves indicate the initialization of visible deformation. Red curves highlight the general failure mode at 33% strain. (**a**) S1; (**b**) S2; (**c**) S3; (**d**) S4; (**e**) S5; (**f**) S6; (**g**) S7; (**h**) S8; (**i**) S9; (**j**) S10. The two deformation stages are not at the same scale.

Compressive tests were performed on an Instron 68TM-50 static testing machine (USA) with a 10 kN force cell. The parts were constrained between the compression platens of the testing machine, the lower one being held fixed, while the upper plate was moved at a constant speed of 1.5 mm/min up to a predefined displacement of 10 mm. All samples were tested under the same conditions. Figure 6 shows images taken during testing. Highlighted in yellow are the areas where the initialization of deformation occurred for each individual sample, while the red curves show the general failure mode of the entire structure, in the fully compressed form.

The gyroid, Figure 6a, S1, showed an initialization of the deformation in the central area of the sample, along the entire length of the layer, also encouraged by the 30° angle formed by the walls with the vertical. This was accompanied by a stable, “layered” failure mechanism with significant “barrel”-type lateral deformation. Sample S2, Figure 6b, simulated a re-entrant-type behavior, in which the arrangement under a sharp angle of the walls dictates the location and direction of the deformation, starting also in the central area. In its final state, it presented a shape similar to the gyroid, but with a better regularity and a more pronounced bi-convex shape. Sample S3, Figure 6c, exhibited an initial lateral deformation of the lower zone on the contour of the specimen, due to the lack of support of the cell walls. This also showed a stable failure mode with small asymmetries, determined by the bending of lower cross-sectional areas until the gradual stiffening caused by the walls’ contact. Sample S4, Figure 6d, presented an instability due to the local buckling of the vertically arranged walls, in three different zones, which dictated how the layers “laid” on top of each other, leading to a less predictable macro deformation with more significant asymmetries. In sample S5, Figure 6e, the wall thickness gradient on the height of the sample led to a failure mechanism “in successive layers”, from top to bottom.

The deformation initialization is found in the contact zone of the metamaterial with the upper face of the sandwich structure. The deformation of sample S6, Figure 6f, was also governed by the buckling of the vertical walls, resulting in a final barrel-like shape, similar to S1, but with a more significant deformation in the lower area. The lack of two-way symmetry of topology in S7, Figure 6g, as well as the onset of deformation through the buckling of a wall arranged on an entire layer of the sample, gave rise to a geometry that was less rigid on the right side, thus manifesting a more important deformation in this area and an asymmetry in yield mode. Metamaterial S8, Figure 6h, presented local instabilities on the periphery of the sample, due to the thin zones that were generated by constraining the model to the nominal dimensions. These led to the fractures of the ligaments thus formed, but did not determine an unstable deformation mode, which was similar to the type obtained for sample S2. Configuration S9, Figure 6i, due to the inclination of the constitutive elements of the cells, presented an initialization and a general failure mode given by the oblique contact of the walls, while maintaining its lateral stability, in contrast to the behavior previously obtained in sample S7. Finally, the stochastic-type geometry S10, Figure 6j, presented a deformation initiation zone that is difficult to identify. A type of deformation dominated by axial loads on the struts is observed, which led to the loss of stability and their inevitable rupture. Finally, the repeated fragmentation of the thinner ligaments, both at the sample’s contour and in depth, determined a behavior that was difficult to predict. With the exception of this sample, local failures were a rare phenomenon, due to both the flexibility of the material used and the definition of the topologies through continuous surfaces that do not encourage the localization of efforts.

**Figure 7:**
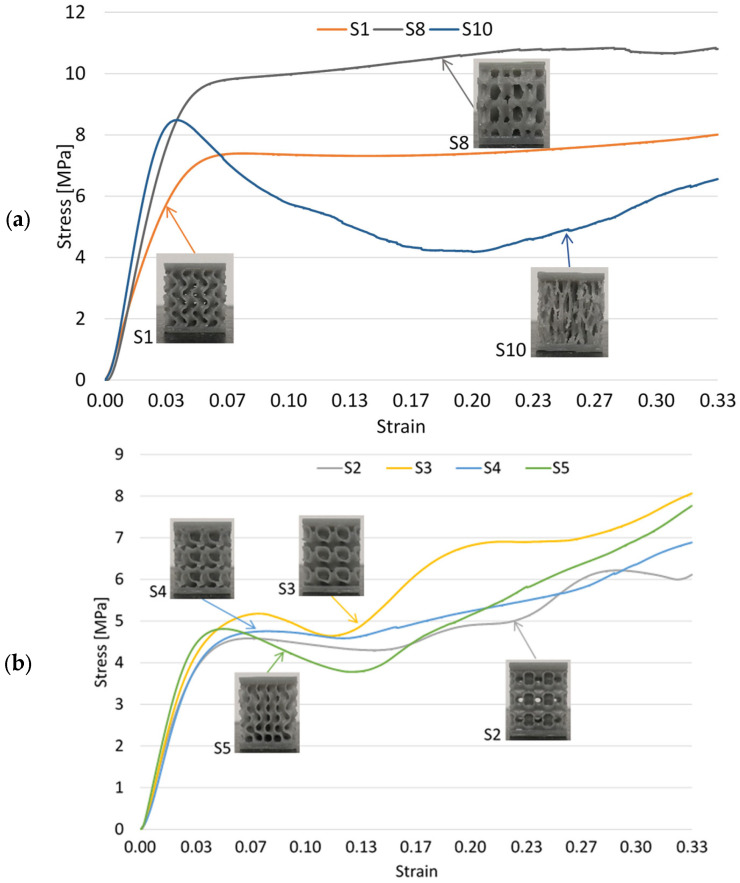

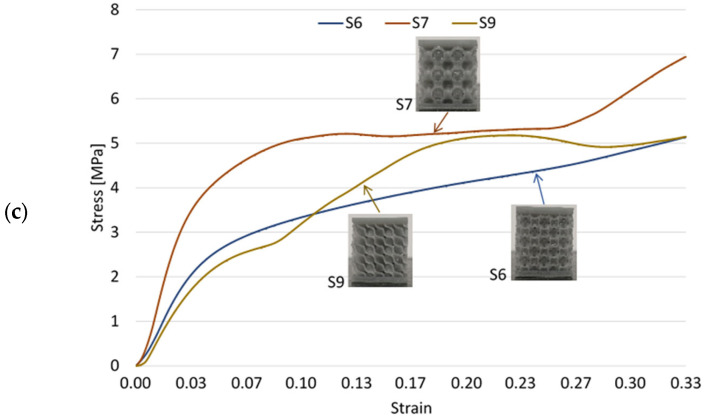
Compressive force versus deformation for samples: (**a**) S1, S8, and S10; (**b**) S2, S3, S4, and S5; (**c**) S6, S7, and S9.

Next, the test results are presented in the form of diagrams of the compressive force as a function of the samples’ deformation. In order to simplify the visualization of the curves of the 10 geometries, they were divided into three categories, depending on the mechanical response obtained after testing. Figure 7a shows the evolution of the gyroid S1, stochastic S10, and S8 samples. S8 and S1 geometries behaved similarly, while the stochastic geometry was included in this comparison due to its different construction from the rest of the samples. A difference in stiffness is observed in favor of the random geometry, but a much less predictable behavior, given by the ruptures of the struts, materialized by the substantial reduction in loading force after moving towards the plastic region. In contrast, the other geometries show a much more stable behavior, without cracks or sudden stiffening due to the contact between adjacent surfaces.

Despite previous results presented in the literature, where stochastic geometries were inferior in all respects to the gyroid structure [6,39,40], it was observed that in terms of maximum loading force and stiffness, they are higher in the case of the randomly defined sample S10 compared to the gyroid S1. However, the behavior dominated by the local buckling of the ligaments led to the premature failure of the samples at relatively low strains and, thus, to a much lower energy absorption capacity.

Geometry S8 also exhibited foam-like behavior, with a long and continuous hardening yield plateau, similar to the response provided by the gyroid sample S1. The force corresponding to the yield point was 31% higher than that recorded for the gyroid, a difference that increased to 34% at a deformation value of 10 mm. After a deformation of 7 mm, the structure starts to show internal cracks, observed in the outer areas of the sample, but which did not lead to premature destruction. Scaling the topology over a larger contour or changing the parameters of the defining mathematical function so that thin walls may not be generated in the peripheral areas will lead to the elimination of this stepwise evolution and a continuous stiffening of the structure, similar to the gyroid geometry.

Figure 7b shows the gradual variation in the loading force for samples S2, S3, S4, and S5. A different response from the case of the classic gyroid was found. In addition to the lower value of up to 40% of the maximum force corresponding to the transition into the plastic domain compared to geometry S1, the force–deformation curves show significant fluctuations in the plateau region under quasi-static compressive loads. This is based on the effect of “softening”, then, respectively, “hardening” of the topology, in steps given by the self-contact between the walls of the cells. It is observed that structures S2, S3, and S5 show a more pronounced weakening behavior, after passing into the plastic deformation zone, while in the case of geometry S4, the value of the capable force increases continuously. The samples displayed in Figure 7b have a more pronounced hardening than the gyroid specimen (Figure 7a), so that the difference between the stress values corresponding to a 33% strain are 27.6% (S2), 0.01% (S3), 3.22% (S5), and 15.29% (S4).

Figure 7c shows the evolution of samples S6, S7, and S9. A continuous stiffening is observed in the case of samples S6 and S7 built from semi-closed cells, attributed to the small-sized constructive features that are formed within the samples. These lead to the buckling of a greater number of walls, and implicitly to more faces coming into contact with each other, before the sample exhibits noticeable “softening”. A similar behavior was also shown by sample S9, built by overlapping the layers under different inclinations, due to the resulting deformation in the diagonal direction.

### 3.2. Influence of Relative Density

In order to estimate how the relative density influences the mechanical properties of cellular metamaterials, the model proposed by Gibson-Ashby in [41] for open cell foams was used, defined by the following equations:

(10) $$\frac{E}{E_{0}}=C_{1}{\left(\frac{\rho }{\rho_{0}}\right)}^{\alpha_{1}}$$

(11) $$\frac{\sigma }{\sigma_{0}}=C_{2}{\left(\frac{\rho }{\rho_{0}}\right)}^{\alpha_{2}}$$

where *E*, *σ*—elastic modulus and yield strength of the proposed structure; *E*_0_, *σ*_0_—elastic modulus and yield strength corresponding to the homogenous structure made from the same material incorporating the entire domain; *C*_1_, *C*_2_-—proportionality constants; *ρ*/*ρ*_0_—relative density of the structure; *α*—fixed exponent whose value depends on the behavior of the structure: *α*_1_ = 2 and *α*_2_ = 1 if the structure has an “ideal bending dominated” response (structures S1, S6, S7, S8) and *α*_1_ = 1 and *α*_2_ = 1 for an “ideal stretching dominated” behavior (S10) [42]. Considering that the model does not include mixed behaviors, *α*_1_ = 1.5 was considered for a such a response (structures S2, S3, S4, S5, S9).

The relative elastic modulus and yield strength for different relative densities of the proposed samples can be predicted by particularizing the previous relationship. The coefficients *C*_1_, used for relative elastic modulus, and *C*_2_, used for relative yield strength, are determined by using the aforementioned formulas, customized with the values of the parameters determined experimentally following the quasi-static compression, for the relative density of 0.3 established throughout this study. These values, presented in Table 2, are used to estimate the modulus of elasticity and the yield stress of the samples at different values of relative density. Due to the complex 3D geometry, the yield stress of each individual sample was approximated by the ratio between the force corresponding to the onset of yielding and the average cross-section of the sample.

Analyzing the obtained *C*_1_ and *C*_2_ coefficients, it is observed that most of them are within the range proposed in the Gibson–Ashby model, except for the value of *C*_2_ corresponding to sample S8, which exceeds the mentioned limit a little bit. This can be attributed to the differences in behavior introduced by the parameters of the printing process, as mentioned by Ravichander et al. in [43], given the empirical nature of the specified limits.

With the help of the determined values, graphs that estimate the behavior of the samples at different values of relative density were constructed. They are divided according to the behavior they exhibited: “bending-dominated”, “stretching-dominated”, or mixed.

**Figure 8:**
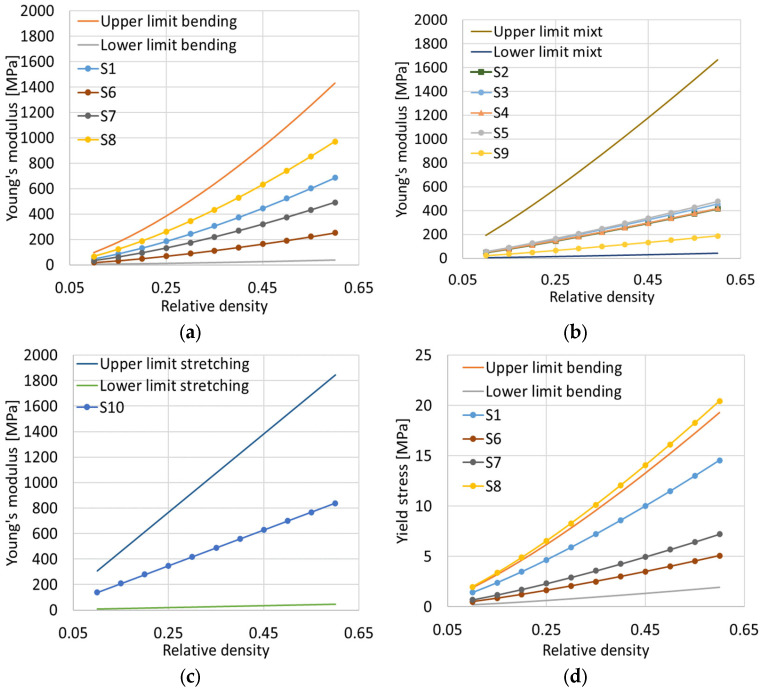

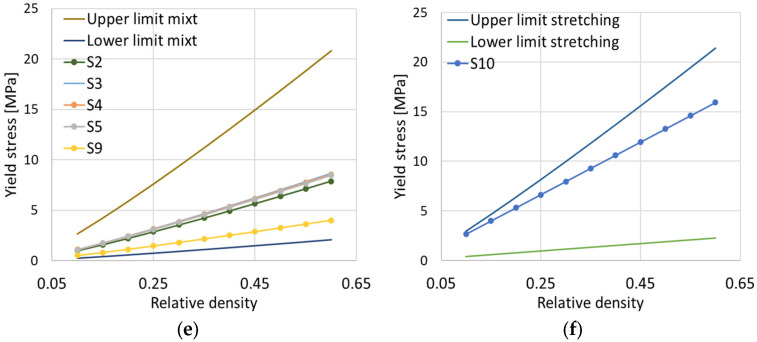
Young’s modulus as a function of relative density for samples that exhibit (**a**) “bending-dominated” behavior; (**b**) “mixed” behavior; (**c**) “stretching-dominated” behavior. Yield stress as a function of relative density for samples that exhibit (**d**) “bending-dominated” behavior; (**e**) “mixed” behavior; (**f**) “stretching-dominated” behavior.

It can be seen in Figure 8a that the gyroid structure is located in the middle of the proposed range, with sample S8 having a slightly higher variation, while samples S6 and S7 show lower values. It is also observed, within the mixed behavior, that the values for both the modulus of elasticity and the yield stress are distributed towards the lower limit. Sample S10, the only one that showed “stretching-dominated” behavior, is also well defined in the range proposed by the considered model. As mentioned previously, S8 has an unexpected behavior since the yield limit exceeds the upper limit, as seen in Figure 8d. A more detailed experimental analysis is needed and will be employed in a future study, in order to validate that the proposed model is well suited for polymer TPMS structures, and that the estimated results are well fitted within the reference limits, taking into account the proportional constants used.

**Figure 9:**
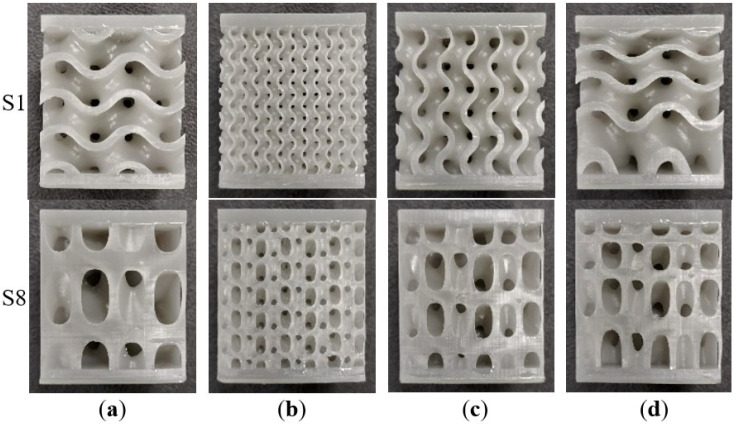
Geometry variations for S1 and S8: (**a**) 2 × 2 × 2 RVE; (**b**) 4 × 4 × 4 RVE; (**c**) wall thickness grading along height; (**d**) geometry grading along height.

After the uniform cell distribution samples were analyzed, variations from the base geometries were architected and tested, in order to have an indication of improvement possibilities. Thus, for the most promising samples, the gyroid S1 and sample S8, new topologies were generated. These can be identified in Figure 9. The relative density was kept constant, with the following alterations: (a) domain filled with 2 × 2 × 2 cells; (b) domain filled with 6 × 6 × 6 cells; (c) wall thickness varied along the height of the structure; (d) cell height decreased towards the upper surface of the sample.

**Figure 10:**
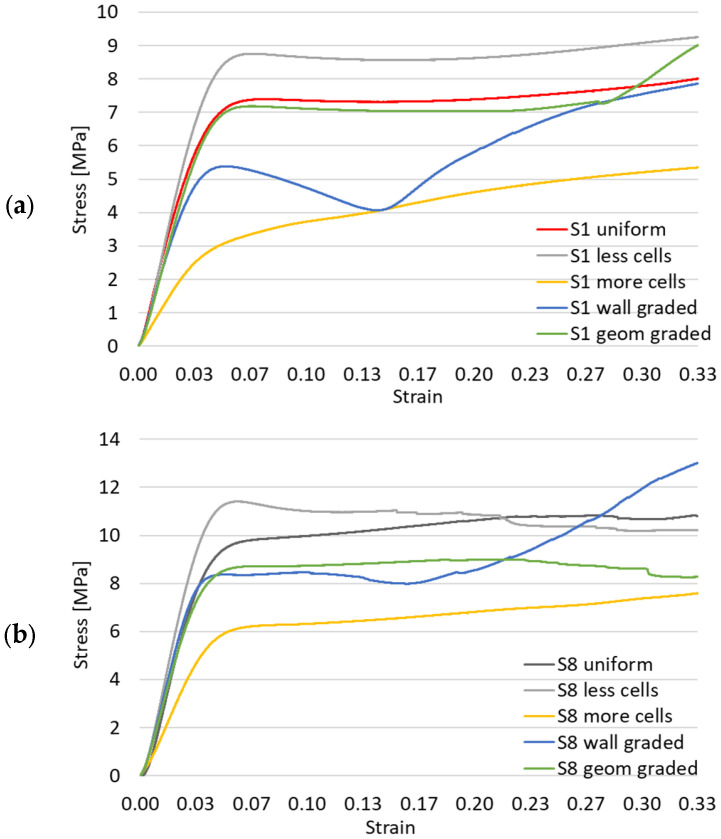
Compressive force as a function of deformation for variations from the basic geometry for (**a**) S1; (**b**) S8.

Figure 10 displays the compressive force as a function of deformation for the modified gyroid and S8 samples. As can be noticed, using more cells does not result in better loading capacity, while having less cells slightly improves the mechanical response. The deformation modes remain unchanged for samples with constant wall thickness. When this is modified, the behavior changes, to a layer-by-layer compression with lower yield stress and gradual stiffening. Modifying the geometry so as to include cells that vary in height along the thickness of the sandwich structure results in a similar response, with a slightly lower force–deformation evolution.

## 4. Discussion and Conclusions

An implicit modeling approach was used to generate novel TPMS structures, whose printability, anisotropy, and mass distribution were analyzed. Multiple samples were successfully fabricated using an SLA method, with no visible defects identified.

Following uniaxial compression tests, the proposed structures presented different methods of deformation. Samples S1, S6, S7, and S8 exhibited a “bending-dominated” behavior characterized by a long plateau with negligible material softening and reduced loading stress fluctuations. Samples S2, S3, S4, S5, and S9 showed a “mixed” behavior, “bending-dominated” and “stretching-dominated”, where yielding is followed by a “softening” zone as the loading force decreases due to local buckling of the walls, until contact between them generates a new stiffening response. Mass distribution inside the sample leads to different cross-sectional area variation along the height of the sample, affecting the general deformation mechanism and energy absorption capacity. The stochastic structure exhibited a purely “stretching-dominated” behavior, where the axial forces in the struts of the specimen led to rapid buckling after the elastic region, leading to a much more pronounced reduction in loading capacity and energy absorption. Compared to the gyroid evolution, the stochastic structure exhibited superior stiffness in the elastic domain and an approximately 6% higher yield strength. The results thus highlight the differences between the two types of 3D metamaterials and manage to assign them a predefined type of deformation in accordance with the literature research [6,44].

Compared to the gyroid structure S1, sample S8 showed a similar loading curve, with values up to 31% higher for the force corresponding to the yield limit and a higher densification rate. The surplus of capable force suggests that an equivalent geometry with a lower relative density could lead to similar values to those obtained for the gyroid structure, but with the advantage of mass reduction.

Starting from the established experimental data, the Young’s modulus and yield stress for different relative density values were estimated. The variations are within the limits proposed in relevant literature, apart from sample S8, which exceeds the upper limit. Given the empirical determination of the limits, advancements in additive manufacturing processes, and different materials used in lattice and TPMS structures, a refining of the reference values for coefficients *C*_1_ and *C*_2_ is required.

Alterations to the uniform gyroid S1 and sample S8 were made in order to see if the compressive behavior could be improved. The same specimen with fewer cells displayed a superior loading curve, while more cells generally resulted in lower stiffness and yield stress. A variation in wall thickness along the height of the sample resulted in a layer-by-layer deformation with gradual stiffening. Modifying the geometry so as to gradually decrease the cell height towards the upper part of the sample resulted in a slightly lower loading curve compared to the uniform specimen.

The study demonstrates that an implicit modeling method can easily generate novel thin-walled TPMSs and stochastic structures, which can have superior properties to already mature topologies, such as the gyroid. Plus, a deformation mechanism was attributed to each sample, and methods of simple and effective parametrization of such topologies were suggested. In general, the stable and predictable response of shell TPMS structures makes them promising candidates for multifunctional applications.

## Figures and Tables

**Table 1:** Mathematical formulation of the proposed topologies.

Sample	Sheet Network Lattice Implicit Function	Equation
S1	*f*(*x*,*y*,*z*) = cos (*x*) × sin (*y*) + cos (*y*) × sin (*z*) + cos (*z*) × sin (*x*)	(1)
S2	*f*(*x*,*y*,*z*) = 2 × [cos (*x*) × cos (*y*) + cos (*y*) × cos (*z*) + cos (*z*) × cos (*x*)] − [cos (2*x*) + cos (2*y*) + cos (2*z*)]	(2)
S3	*f*(*x*,*y*,*z*) = cos (*x*) × cos (*y*) + cos (*x*) × cos (*z*) + cos (*y*) × cos (*z*) + sin (*x*) × cos (*y*) + sin (*x*) × cos (*z*)+ sin (*y*) × cos (*z*) + sin (*y*) × cos (*z*) + sin (*z*) × cos (*x*) + sin (*z*) × cos (*y*)	(3)
S4	*f*(*x*,*y*,*z*) = cos (2*x*) × cos (*y*) × cos (*z*) + cos (2*y*) × cos (*x*) × cos (*z*) + cos (2*z*) × cos (*x*) × cos (*y*) + sin (*x*) × cos (*y*) + sin (*x*) × cos (*z*) + sin (*y*) × cos (*z*) + sin (*y*) × cos (*z*) + sin (*z*) × cos (*x*) + sin (*z*) × cos (*y*)	(4)
S5	*f*(*x*,*y*,*z*) = sin (*x*) × cos (*y*) × *z*/2 + sin (*y*) × cos (*z*) × *x*/2 + sin (*z*) × cos (*x*) × *y*/2	(5)
S6	*f*(*x*,*y*,*z*) = 4 × cos (*x*) × cos (*y*) × cos (*z*) − [cos (2*x*) × cos (2*y*) + cos (2*y*) × cos (2*z*) + cos (2*z*) × cos (2*x*)]	(6)
S7	*f*(*x*,*y*,*z*) = 4 × sin (*x*) × cos (*y*) × cos (*z*) − [cos (*x*) × cos (*y*) + cos (*y*) × cos (*z*) + cos (*z*) × cos (*x*)]	(7)
S8	*f*(*x*,*y*,*z*) = 8 × cos (*x*/2) × cos (*z*/2) × sin (*x*/2)+ 8 × cos (*y*/2) × cos (*z*/2) × sin (*y*/2) + 8 × cos (*x*/2) × cos (*y*/2) × sin (*z*/2)	(8)
S9	*f*(*x*,*y*,*z*) = sin (*x*) × sin (*y*) + sin (*x*) × sin (*y*) + sin (*x*) × sin (*y*) − 4 × cos (*x*) × cos (*y*) × cos (*z*)	(9)

**Table 2:** Gibson–Ashby coefficient values for the proposed structures.

	Reference Values [41]	S1	S2	S3	S4	S5	S6	S7	S8	S9	S10
*C*_1_	0.1–4	1.92	1.43	1.56	1.44	1.64	0.71	1.37	2.72	0.65	1.82
*C*_2_	0.1–1	0.75	0.38	0.42	0.41	0.41	0.26	0.37	1.06	0.19	0.71

## Data Availability

The raw data supporting the conclusions of this article will be made available by the authors on request.
